# Stones, Bones, Groans, and Psychic Moans: Primary Hyperparathyroidism Presenting as Surgical Emergency

**DOI:** 10.7759/cureus.4989

**Published:** 2019-06-25

**Authors:** Maryam Saleem, Hassaan Iftikhar

**Affiliations:** 1 Internal Medicine, Waterbury Hospital, Waterbury, USA; 2 Internal Medicine, St. Francis Medical Center, Seton Hall University, Trenton, USA

**Keywords:** peptic ulcer disease, hyperparathyroidism, hypercalcemia, kidney stones

## Abstract

Primary hyperparathyroidism (PHPT) is the third most common endocrine disorder after diabetes and thyroid disease. Most cases of hyperparathyroidism remain clinically silent. The clinical manifestations of hypercalcemia captured in the classic medical student mnemonic of “stones, bones, groans, and psychic moans” are often not found. Sometimes patients can present with unique complications. This case describes perforated peptic ulcer as the first presentation of primary hyperparathyroidism.

## Introduction

Primary hyperparathyroidism (PHPT) occurs in about 1% of the adult US population [1]. The diagnosis is usually established by routine biochemical testing as patients remain symptom-free initially. Peptic ulcer disease due to hyperparathyroidism is rare and has been described in only a few case reports [2-4]. Most patients with PHPT are cured with parathyroidectomy. We present a patient who was admitted for perforated peptic ulcer and found postoperatively to have symptomatic hypercalcemia with multiple organ involvement from recurrent hyperparathyroidism.

## Case presentation

**Figure 1:**
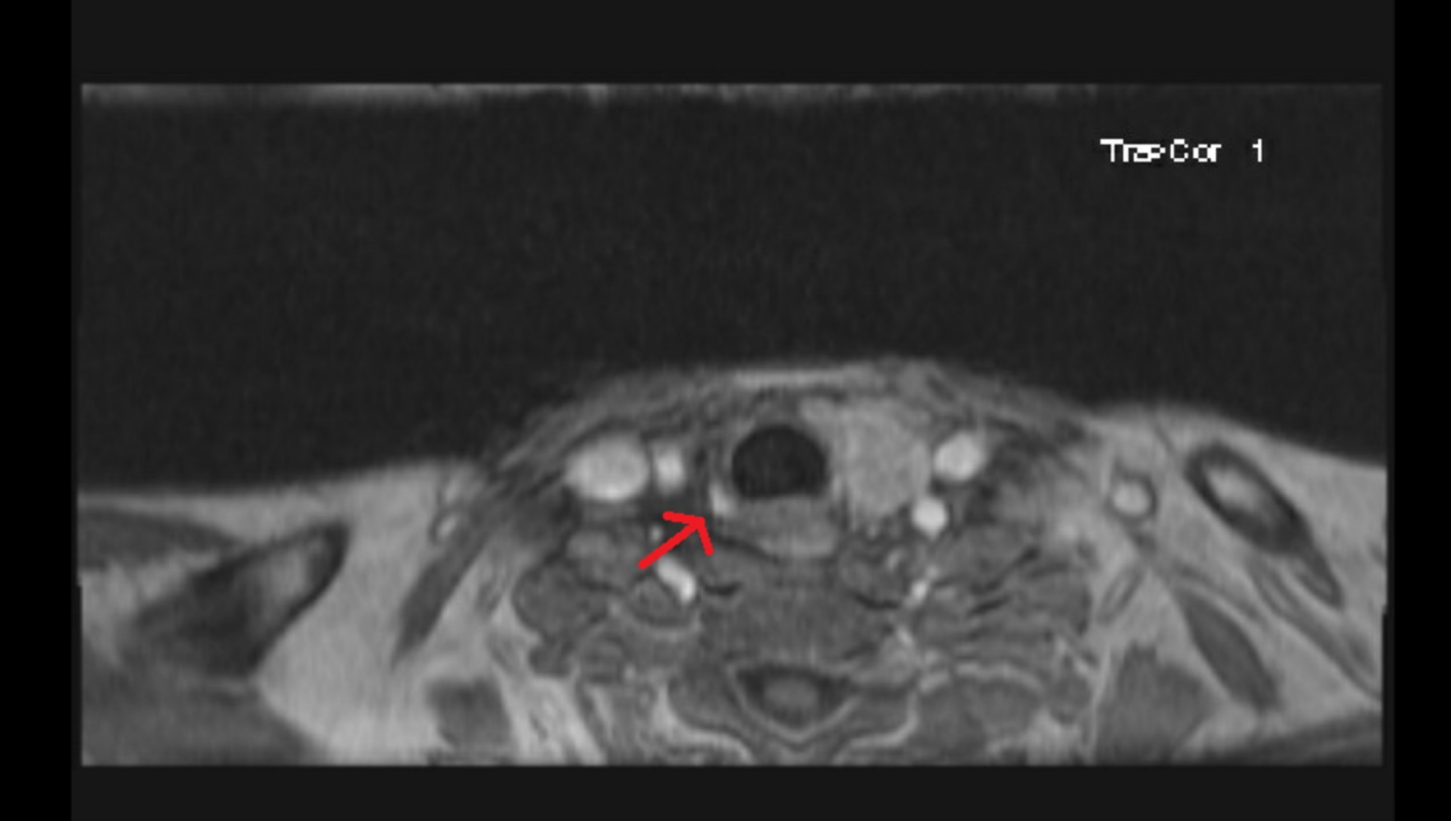
MRI of the neck with contrast showing an arterially enhancing nodule (arrow) in the region of the right thyroid resection bed suggestive of parathyroid adenoma.

A 46-year-old male with a past medical history of right thyroidectomy, chronic kidney disease stage II, nephrolithiasis, gout, and venous thromboembolism presented with abdominal pain secondary to a perforated peptic ulcer. He underwent surgical repair and postoperatively developed acute kidney injury, acute gout, and recurrent kidney stones prompting medical consultation. His workup showed an elevated serum calcium level of 13 mg/dL(normal: 8.5-10.2 mg/dL). With such a past medical history, the patient himself was not aware of any known diagnosis of hypercalcemia and there had been no previous laboratory values to compare with. Further workup of hypercalcemia revealed an elevated parathyroid hormone level (PTH) of 158 pg/mL (normal: 10-65 pg/mL) with a low vitamin D (25 hydroxy) level of 8 ng/mL (normal: 20 ng/mL-50 ng/mL). He was treated with intravenous fluids, cinacalcet, and pamidronic acid. The patient underwent MRI of the neck (Figure 1).

Magnetic resonance imaging of the neck confirmed a diagnosis of parathyroid adenoma. Post-discharge his calcium level trended up from a new baseline of 10 mg/dL to 12.5 mg/dl, and the PTH level rose to 313 pg/mL. He underwent re-operative parathyroidectomy with resection of the right parathyroid gland. Pathology confirmed atypical enlarged parathyroid tissue. Postoperatively, his calcium and PTH levels normalized.

The etiology of peptic ulcer disease was likely hypercalcemia. Other differentials were also considered including Zollinger-Ellison syndrome but his gastrin levels were normal. Multiple endocrine neoplasia (MEN1) was also a consideration but the patient didn’t have any signs of prolactinoma and there was no evidence of pancreatic mass to suggest pancreatic tumors as per initial abdominal imaging. Pathology of the gastric specimen after surgery was negative for *Helicobacter pylori.*

## Discussion

Our patient illustrated the full spectrum of disease and classical manifestations of primary hyperparathyroidism, which is very uncommon in the United States. The disease is considered clinically silent and diagnosis is usually on routine biochemical tests. Most people present with fatigue and tiredness. Other common symptoms at the time of presentation include renal stones, bone involvement, and impaired cognition [1]. According to early literature, the incidence of peptic ulcer disease in patients with hyperparathyroidism is 9% [5]. Peptic ulcer disease is less common now because of proton pump inhibitors. Symptoms usually resolve after the surgery though studies have shown that recurrence of hyperparathyroidism is not uncommon (7%-8% in 10 year follow-up) but it may occur more than 20 years after treatment [6-8]. Our patient illustrates that it is important to look beyond the initial presentation of any surgical emergency to identify the underlying cause of disease for definitive treatment. A multidisciplinary team approach is the best option in this setting.

## Conclusions

Peptic ulcer disease is a well-known complication of untreated PHPT; however, cases of a perforation of peptic ulcer as the first clinical manifestation of primary hyperparathyroidism are extremely rare. Diagnosis of PHPT is usually biochemical. Asymptomatic hypercalcemia without any signs or symptoms should raise the concern for hyperparathyroidism. Early symptoms are usually nonspecific and include fatigue, constipation, weakness, and bone and muscle pain. Diagnosis of PHPT is usually established by measuring PTH and calcium levels. 25 hydroxy vitamin D level is often measured to distinguish PHPT from secondary hyperparathyroidism. Our patient illustrates that it is important to look beyond the initial presentation of any surgical emergency to identify the underlying cause of disease for definitive treatment. A multidisciplinary team approach is the best option in this setting.

## References

[1] Madkhali T, Alhefdhi A, Chen H, Elfenbein D (2016). Primary hyperparathyroidism. Turkish J Surg.

[2] Ahmad M, Vaidyan P, Ahmeda A (1998). An uncommon aetiology of perforated gastric ulcer. BMJ.

[3] Efremidou EI, Liratzopoulos N, Papageorgiou MS, Karanikas M, Pavlidou E, Romanidis K, Manolas KJ (2007). Peptic ulcer perforation as the first manifestation of previously unknown primary hyperparathyroidism. Case Rep Gastroenterol.

[4] Xie D, Hu K, Xian Y (2018). A life-threatening duodenal ulcer hemorrhage due to previously unknown primary hyperparathyroidism. Gastroenterol Rep.

[5] Ebert EC (2010). The parathyroids and the gut. J Clin Gastroenterol.

[6] Hedbäck G, Odén A (2003). Recurrence of hyperparathyroidism; a long-term follow-up after surgery for primary hyperparathyroidism. Eur J Endocrinol.

[7] Linos DA, van Heerdan JA, Abboud CF, Edis AJ (1978). Primary hyperparathyroidism and peptic ulcer disease. Arch Surg.

[8] Gopinath P, Sadler GP, Miha R (2010). Persistent symptomatic improvement in the majority of patients undergoing parathyroidectomy for primary hyperparathyroidism. Langenbecks Arch Surg.

